# Abnormal Alterations of Regional Spontaneous Neuronal Activity in Inferior Frontal Orbital Gyrus and Corresponding Brain Circuit Alterations: A Resting-State fMRI Study in Somatic Depression

**DOI:** 10.3389/fpsyt.2019.00267

**Published:** 2019-04-30

**Authors:** Rui Yan, ShiWan Tao, HaiYan Liu, Yu Chen, JiaBo Shi, YuYin Yang, RongXin Zhu, ZhiJian Yao, Qing Lu

**Affiliations:** ^1^Department of Psychiatry, The Affiliated Brain Hospital of Nanjing Medical University, Nanjing, China; ^2^Nanjing Brain Hospital, Medical School of Nanjing University, Nanjing, China; ^3^School of Biological Sciences and Medical Engineering, Southeast University, Nanjing, China; ^4^Child Development and Learning Science, Key Laboratory of Ministry of Education, Nanjing, China

**Keywords:** somatic depression, resting-state functional magnetic resonance imaging, amplitude of low-frequency fluctuation, functional connectivity, limbic-cortical network, default mode network

## Abstract

**Background:** Major depressive disorders often involve somatic symptoms and have been found to have fundamental differences from non-somatic depression (NSD). However, the neural basis of this type of somatic depression (SD) is unclear. The aim of this study is to use the amplitude of low-frequency fluctuation (ALFF) and functional connectivity (FC) analyses to examine the abnormal, regional, spontaneous, neuronal activity and the corresponding brain circuits in SD patients.

**Methods:** 35 SD patients, 25 NSD patients, and 27 matched healthy controls were selected to complete this study. The ALFF and seed-based FC analyses were employed, and the Pearson correlation was determined to observe possible clinical relevance.

**Results:** Compared with NSD, the SD group showed a significant ALFF increase in the right inferior temporal gyrus; a significant ALFF decrease in left hippocampus, right inferior frontal orbital gyrus and left thalamus; and a significant decrease in the FC value between the right inferior frontal orbital gyrus and the left inferior parietal cortex (*p* < 0.05, corrected). Within the SD group, the mean ALFF value of the right inferior frontal orbital gyrus was associated with the anxiety factor scores (r = –0.431, *p* = 0.010, corrected).

**Conclusions:** Our findings suggest that abnormal differences in the regional spontaneous neuronal activity of the right inferior frontal orbital gyrus were associated with dysfunction patterns of the corresponding brain circuits during rest in SD patients, including the limbic-cortical systems and the default mode network. This may be an important aspect of the underlying mechanisms for pathogenesis of SD at the neural level.

## Introduction

A major depressive disorder (MDD) is characterized by the presence of a depressive mood, a loss of interest or pleasure, psychomotor changes, guilt, worthlessness and sleep abnormalities, and it is one of the most common mood disorders (1). An MDD that is accompanied by somatic symptoms, including fatigue, appetite and sleep disturbance, was defined by Silverstein as somatic depression (SD) (2). It has been found that SD differs from non-somatic depression (NSD) in regards to the gender ratio (3–5), developmental patterns (6), and awareness of behavioural errors (7). In addition, in the Star*D study, the response to biological-based treatments, including Citalopram, Sertraline, and others, was better for NSD than for SD (8). Thompson and Bland found that the SD hypothesis can only account for a relatively small amount of the depression variance (9). One interpretation is that SD may be rooted in psychosocial forces while NSD may be rooted more strongly in genetic and endogenous forces (6). In individuals with MDD, the somatic symptoms appear to be maintained even in the absence of explicit environmental stimuli. The perpetuation of SD by the brain is still not well understood. However, Geng et al. found that SD exhibits abnormal regional homogeneity in the frontal and temporal regions (5). Thus, the somatic-related differences in MDD may be linked to a stronger neurobiological diathesis. Therefore, functional neuroimaging offers potential insights into the associated neural mechanisms.

Since the study by Biswal et al. (10), resting-state functional magnetic resonance imaging (fMRI) has become an increasingly popular technique to study mental diseases. In particular, it has been widely employed to investigate the neuropathology of depression. From previous reports, it was found that depressed patients exhibit abnormal activation in the cortical or limbic regions (11–13). A meta-analysis of MDD revealed alterations in the spontaneous activity of multifocal brain areas in MDD subjects compared to healthy controls, including in the dorso-lateral prefrontal cortex, superior frontal gyrus, orbitofrontal cortex, superior and middle temporal gyrus, insula, precuneus, striatum, thalamus, precuneus, posterior cingulate cortex, hippocampus and cerebellum (11). Previous studies also suggested the presence of dysfunctions in brain networks of SD patients, such as the default mode network (DMN), the ventromedial prefrontal network, the left fronto-parietal and right fronto-parietal networks, the default network, the salience network (14) and the prefrontal-limbic-thalamic networks (15, 16). Both these results imply that multiple network circuit dysregulations exist in patients with depression.

Previous fMRI studies suggest that there are several associations between somatic symptoms and certain brain region abnormalities. Sleep disturbances are linked to activity in the corticolimbic circuitry, such as the prefrontal cortex, amygdala, striatum, insular cortex and thalamus (17, 18). Insomnia symptoms are also associated with posterior DMN (19), fatigue is associated with the medioventral occipital cortex and precentral gyrus (20), while pain is linked to activity in the left putamen, left frontal gyrus and right insula (20).

One hypothesis is that dysfunctions of sensitivity, awareness, and attention play important roles in the somatic symptoms of depression (21). Negative internal bodily stimuli can amplify the perceived intensity of pain and other somatic sensations and have been related to the anterior cingulate cortex and prefrontal cortex (22). In summary, we found that brain abnormalities in depressed patients with somatic symptoms were not exactly the same as those in NSD patients. In addition, patients with somatic symptoms show that brain region abnormalities were localized in the limbic-cortical systems.

Although the above studies have revealed that regional and inter-regional abnormalities exist in certain brain areas of depressed patients during the resting-state, they failed to assess the alterations in both regional spontaneous neuronal activity and the corresponding brain circuits during rest. In other words, one brain region may exhibit abnormal functionality from its connectivity with other regions but may not necessarily be abnormal itself. Similarly, abnormal regional activity may not imply abnormal connectivity between it and other regions. In addition, less attention has been given to SD patients with respect to fMRI. Therefore, it is of interest and meaningful to explore the underlying abnormal regional activity and its corresponding brain circuits in SD patients.

It was reported by Zang et al. (23) that ALFF can be used to examine regional brain activities in rest-state fMRI, since ALFF is strongly correlated to the BOLD signal time courses across different regions (24, 25). Since then, it has been found that MDD patients have altered ALFF in a number of emotional or cognitive-related brain areas, including parts of the frontal, temporal, parietal, and occipital cortices and the cerebellum (3, 26–28). As an additional advantage, ALFF can be used to investigate the neuronal activity of the entire brain (25) and select the “seed” voxels for further functional connectivity (FC) analysis, which avoids potential bias (29). FC analyses, which are based on the temporal correlations between spatially remote neurophysiological events, are a method used for measuring the correlation coefficients of all brain areas with a single pre-defined region. Recent studies of brain networks using an FC analysis have provided novel insights into how distributed brain regions are functionally integrated in MDD patients (30). Seed-based FC analyses could be a promising approach to observe the connectivity between abnormal regions and other regions in SD patients.

The ALFF and FC analyses have been combined and employed with regards to several brain disorders to provide additional important information and understanding of the considered diseases, such as migraines (31), heroin addiction (32), unmedi­cated depression (33), and NSD (12). However, to the best of our knowledge, less attention has been given to the application of this method to first-episode SD patients. On the other hand, Fan et al. (34) demonstrated that functional connectivity during the resting-state was modulated by autonomic arousal. In addition, Di X et al. (35) found that within-network connections were correlated to the local fluctuation amplitudes, and the dynamic network-functional connectivity properties were associated with the intrinsic activities of the brain networks (36). Thus, we hypothesize that the difference in the intrinsic activities of limbic-cortical regions may be associated with the functional connectivity in the brain circuits of SD patients.

In this study, we first used ALFF measurements to investigate the regional spontaneous neuronal activity in SD and NSD patients, and then identified which brain regions from the different ALFFs were regions of interest (ROIs). After that, we applied a seed-based FC analysis to elucidate brain circuit spontaneous neuronal activity properties. We hypothesize that abnormal ALFF and FCs would be discovered in certain emotional and cognitive-related brain areas in SD patients. Meanwhile, we also hypothesize that alterations in brain function would be associated with the severity of SD.

## Methods

### Subjects

From May 2011 to August 2015, we recruited 60 patients who underwent the first episode of depression from the Department of Psychiatry of the Affiliated Brain Hospital of Nanjing Medical University. Meanwhile, 27 healthy controls (HCs) who were matched in age, education, and gender (mean age ± standard deviation = 32.22 ± 7.75 years old, mean education ± standard deviation = 14.92 ± 1.77 years, 13 males) were recruited. The inclusion criteria included being right-handed, aged 20-45, and Chinese Han. The Mini International Neuropsychiatric Interview (MINI) (37) was used to confirm MDD diagnoses by two qualified psychiatrists (Dr Yu Chen and Dr JiaBo Shi) according to DSM-IV-TR criteria (1). Before the MRI scan, all patients were required to have at least a 2-week medication washout period. The total score of the 17-item Hamilton Rating Scale for Depression (HRSD) (38) had to be ≥ 17 on the scanning day. All patients had no somatic disease, head injury history, or any other additional psychiatric disease. Each HC was filtered using the non-patient version of the Structured Clinical Interview from the DSM-IV-TR, and none had any medical, neurological, or psychiatric illness, or had a first-degree-relative family history of major psychiatric or other neurological illness.

The patients were divided into SD and NSD groups in accordance with the criteria described by Silverstein (2). The SD patients conformed to at least three of the following symptoms: regular severe headaches, previous or current trouble breathing for no apparent reason, frequent staying asleep without waking up or trouble falling asleep at night, continual unexplained fatigue, and a poor body image/preference for thinness and/or eating disorders (2). The patients were aged from 20 to 45 years old (mean age ± standard deviation = 33.08 ± 8.92 years old, mean education ± standard deviation = 13.6 ± 3.3 years, 19 males). The NSD patients were defined as MDD but were unaccompanied by sufficient somatic symptoms. These patients were aged from 20 to 45 years old (mean age ± standard deviation = 33.1 ± 8.4 years old, mean education ± standard deviation = 14.8 ± 3.3 years, 11 males).

This study was approved by the Research Ethics Review Board of the Affiliated Brain Hospital of Nanjing Medical University. Signed informed consent was obtained after a complete description of the study was given to all subjects.

### MRI Scan Acquisitions

Imaging data were obtained with an 8-channel radio frequency coil on a 3-Tesla Siemens verio scanner at the Affiliated Brain Hospital of Nanjing Medical University on the recruitment day. The heads of all subjects were placed in a birdcage coil and fit with foam padding to reduce head motion. Participants were required to remain motionless and relaxed and to keep their eyes closed. The parameters for T1 anatomic axial imaging were as follows: repetition time (TR) = 1900 ms, echo time (TE) = 2.48 ms, flip angle (FA) = 9°, number of slices = 176, slice thickness = 1 mm, in plane voxel resolution = 1 mm × 1 mm, and field of view (FOV) = 25 × 25 cm^2^. Resting-state fMRI data were obtained through the use of an echo-planar imaging (EPI) sequence. The parameters were as follows: TE = 40 ms, TR = 3000 ms, FA = 90°, slice thickness = 4 mm, slice gap = 4 mm, number of slices = 32, FOV = 24 × 24 cm^2^, matrix size = 64 × 64, in plane voxel resolution = 3.75 mm × 3.75 mm, and 133 volumes.

### Data Pre-Processing

The data processing assistant for resting-state fMRI (DPARSF) was used to perform the standard pre-processing steps (39). The first 6 functional volumes were discarded to allow the participants to get used to the scanner noise and account for T1 saturation effects. Then, slice timing, head-motion correction, and spatial normalization to that of the Montreal Neurological Institute (MNI, resampled voxel size = 3 × 3 × 3 mm^3^) were conducted. Participant head motion should be less than 2 mm translations in any axial direction and 2° in any angular dimension at most. An estimate of the head motion was calculated as the frame-wise displacement (FD) at each time point using 6 displacements from the rigid body motion correction procedure (40). There were no significant differences in the FD values among the three groups (**Table 1**). The structural images were normalized to the structural (T1-weighted) MNI template. No participant was precluded for reasons of excessive head motion or bad normalization. The remaining data were smoothed using a 6 mm full-width at half maximum (FWHM) Gaussian kernel. Finally, a temporal ﬁlter (0.01∼0.08 Hz) and linear detrend were used to reduce the high-frequency noise and low-frequency drift.

**Table 1:** Participant demographic, clinical characteristics and Frame-wise displacement.

Variables	SD (n = 35)	NSD (N = 25)	HC (n = 27)	*p* value
Age (years)	33.1 ± 8.9	33.1 ± 8.4	32.3 ± 7.8	0.926^a^
Gender (male:female)	19:16	11:14	13:14	0.725^b^
Education (years)	13.6 ± 3.3	14.8 ± 3.0	14.7 ± 1.8	0.162^a^
Illness duration (months)	6.8 ± 4.2	8.4 ± 5.6	–	0.224^c^
HRSD score	26.2 ± 4.2	22.1 ± 4.4	2.0 ± 1.9	0.000^a^
Factors in HRSD				
Anxiety	7.3 ± 2.4	5.1 ± 2.2	–	0.000^c^
Weight loss	0.9 ± 0.8	0.8 ± 0.9	–	0.498^c^
Cognitive disturbance	4.1 ± 1.8	3.7 ± 1.9	–	0.399^c^
Retardation	8.1 ± 1.9	8.0 ± 1.8	–	0.838^c^
Sleep disturbance	4.7 ± 1.6	4.4 ± 2.0	–	0.383^c^
FD	0.11 ± 0.04	0.11 ± 0.08	0.11 ± 0.05	0.893^a^

SD, somatic depression; NSD, non-somatic depression; HC, healthy control; HRSD, Hamilton rating scale for depression; FD, frame-wise displacement.

a The p values were obtained with a one-way ANOVA.

b The p values were obtained with chi-square test.

c The p values were obtained with a two-sample t-test.

### ALFF Analysis

The ALFF was calculated using the DPARSF software. The filtered time series was rendered into the frequency domain using a fast Fourier transform (FFT) for each voxel, except for the cerebellum, (taper percent = 0 and FFT length = shortest). The square root was calculated at each power spectrum frequency and the averaged square root at each voxel was acquired across 0.01-0.08 Hz. This averaged square root was regarded as the ALFF value (23). For normalization purposes, the ALFF of each voxel, except the cerebellum, was divided within a brain mask using the mean ALFF value, which was acquired by undocking the tissues outside the brain using the MRIcro software (http://www.psychology.nottingham.ac.uk/staff/cr1/mricro.html). This standardization procedure is consistent with that used in PET studies (41).

### Functional Connectivity Analysis

To prepare for the functional connectivity analysis, several sources of fake variances that included the parameters of head motion, averaged global BOLD signals and mean BOLD signals in the ventricular and white matter regions were removed. We examined the functional connectivity using a seed-based approach. To obtain the ROIs of the seeds, we defined the voxels that had peak regions with significant differences between the SD and NSD groups as the centres, and used each centre to draw a sphere with a 6-mm radius. Then, a correlation analysis was performed between the averaged time series of all voxels in the ROI and the time series of the removed cerebellum from the entire brain template based on the voxel-wise approach. The correlation coefficients were converted into z values using the Fisher’s transformation to improve the normality.

### Statistical Analyses

A one-way analysis of variance (ANOVA) was used to compare the age, education and HRSD-17 scores, while chi-square tests were used for the gender of the SD, NSD and HC groups. The disease duration and the factors from the HRSD-17 were compared between the SD and NSD groups using two-sample t-tests (SPSS 19.0 software, SPSS Inc., Chicago, IL). The level of statistical significance was set at p < 0.05 (two-tailed).

To compare the ALFF or FC values among the three groups, a one-way ANOVA was applied to the resting-state fMRI data analysis toolkit (REST) software after checking the age, sex and years of education as covariates and correcting for multiple comparison. The following *post hoc* t-tests were performed to identify differences between each pair of groups (42). On one hand, to check for multiple comparisons in the statistical analysis of the ALFF values, we used the false discovery rate (FDR) correction to adjust the alpha level (*p* < 0.05 and cluster size of at least 20 voxels, FDR corrected). On the other hand, to check for multiple comparisons in the statistical FC values, we used the Gibbs random fields (GRF) correction to adjust the alpha level. The one-way ANOVA was applied to the 6-mm FWHM parameter, which was combined with the individual voxels *p* < 0.001 with a minimum cluster size of 33 voxels (*p* < 0.05, GRF corrected). Then, *post hoc* t-tests were performed using the parameters to determine the significance threshold, which was combined with the individual voxels given *p* < 0.001 and a minimum cluster size of 7 voxels (*p* < 0.05, GRF corrected). We acquired the parameters using a function of the REST software called the cluster threshold size estimator plug-in (42). To use the parameters, the false probability rate of the corrected family-wise was set at *p* < 0.05.

A Pearson correlation analysis was conducted to observe the possible clinical relevance between alterations in brain function and the severity of somatic symptoms. We used the same method as that for the seed-based FC to obtain the ROIs, which showed significant differences in the FC between the SD and NSD groups. Then, the mean ALFF values or mean z values of the ROIs were calculated for each group subject. Finally, Pearson correlation analyses were performed between the abnormal mean ALFF values or mean z values of the ROIs and the following symptom features: the HRSD total scores and three separate symptomatic factors, including anxiety, weight loss, and sleep disturbance. Bonferroni correction was performed to reduce the rate of false positives for multiple comparisons, where the threshold was set to alpha = 0.05/4.

## Results

### Demographic Results

Clinical, demographic data and the frame-wise displacement of the participants are shown in **Table 1**. The distributions of gender, age, and education were matched among the three groups.

### ALFF: Group Differences

**Figure 1:**
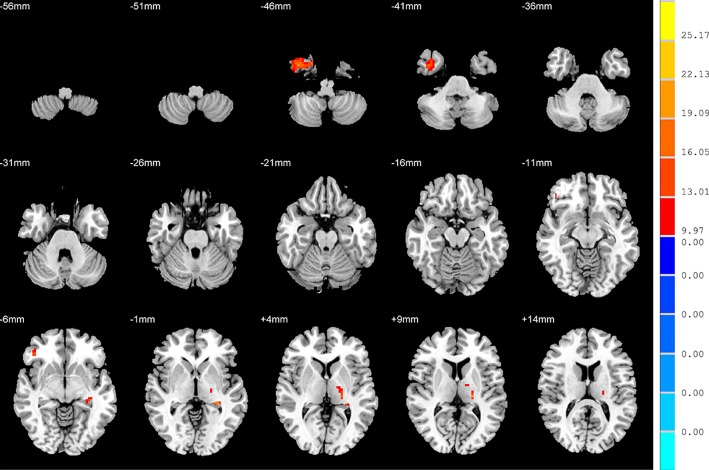
Brain regions showing differences in the amplitudes of low-frequency fluctuations among the three groups with age, sex and years of education as covariates. The colour bar signifies the F-value of the ANOVA analysis with *p* < 0.05 and corrected for multiple comparisons using FDR.

There were widespread differences in the ALFF values between the SD, NSD and HC throughout the right inferior temporal gyrus, left hippocampus, right inferior frontal orbital gyrus and left thalamus from the ANOVA analysis (*p* < 0.05, FDR corrected) (**Table 2** and **Figure 1**).

**Table 2:** Brain areas with amplitudes of low-frequency fluctuation differences among all groups.

Brain regions	MNI (x y z)			Cluster size	F/*t* -value
Three group					
R Inferior temporal gyrus	39	0	–45	64	19.261^a^
L Hippocampus	–27	–33	0	21	20.622^a^
R Inferior frontal orbital gyrus	45	36	–9	20	22.208^a^
L Thalamus	–21	–24	6	31	28.208^a^
SD > NSD					
R Inferior temporal gyrus	39	0	–45	64	5.606^b^
SD < NSD					
L Hippocampus	–21	–36	6	20	−4.852^b^
R Inferior frontal orbital region	45	39	–9	20	−5.765^b^
L Thalamus	–21	–24	6	31	−6.709^b^
NSD > HC					
L Hippocampus	–27	–33	0	21	7.259^b^
R Inferior frontal orbital gyrus	45	36	–9	20	4.864^b^
L Thalamus	–21	–24	6	31	6.069^b^
NSD < HC					
R Inferior temporal gyrus	39	3	45	64	−5.448^b^

ALFF, amplitude of low-frequency fluctuation; MNI, Montreal Neurological Institute; x, y, z, are the coordinates of the primary peak locations in the MNI space; SD, somatic depression; NSD, non-somatic depression; HC, healthy control; F, statistical value of the peak voxel showing the significant ALFF differences among all the groups; t, statistical value of the peak voxel showing significant ALFF differences between the SD and NSD, or NSD and HC (p < 0.05, corrected for FDR correction).; L, left; R, right.

a The F statistical value.

b The t statistical value.

### ALFF: SD versus NSD Patients

**Figure 2:**
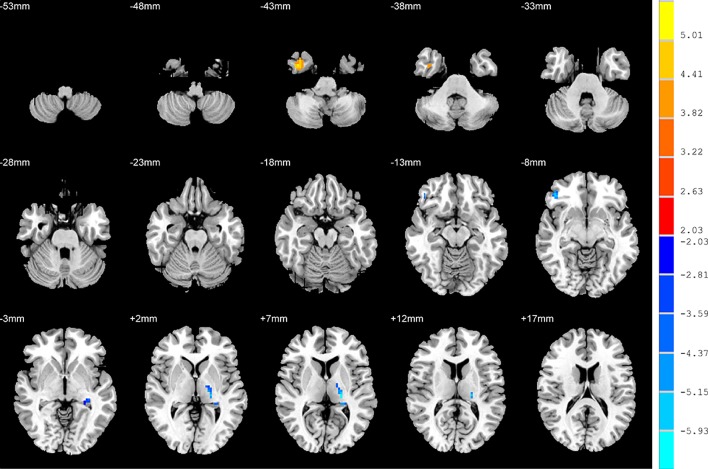
Brain regions showing differences in the amplitudes of low-frequency fluctuations between the SD patients and NSD patients. The colour bar signifies the t-value of the independent t-tests between the two groups with *p* < 0.05 and corrected for multiple comparisons using FDR.

Compared to the NSD patients, the SD patients showed a significant ALFF increase in the right inferior temporal gyrus and significant decreases in the left hippocampus, right inferior frontal orbital gyrus and left thalamus (*p* < 0.05, FDR corrected) (**Table 2** and **Figure 2**).

### ALFF: NSD Patients versus HC

**Figure 3:**
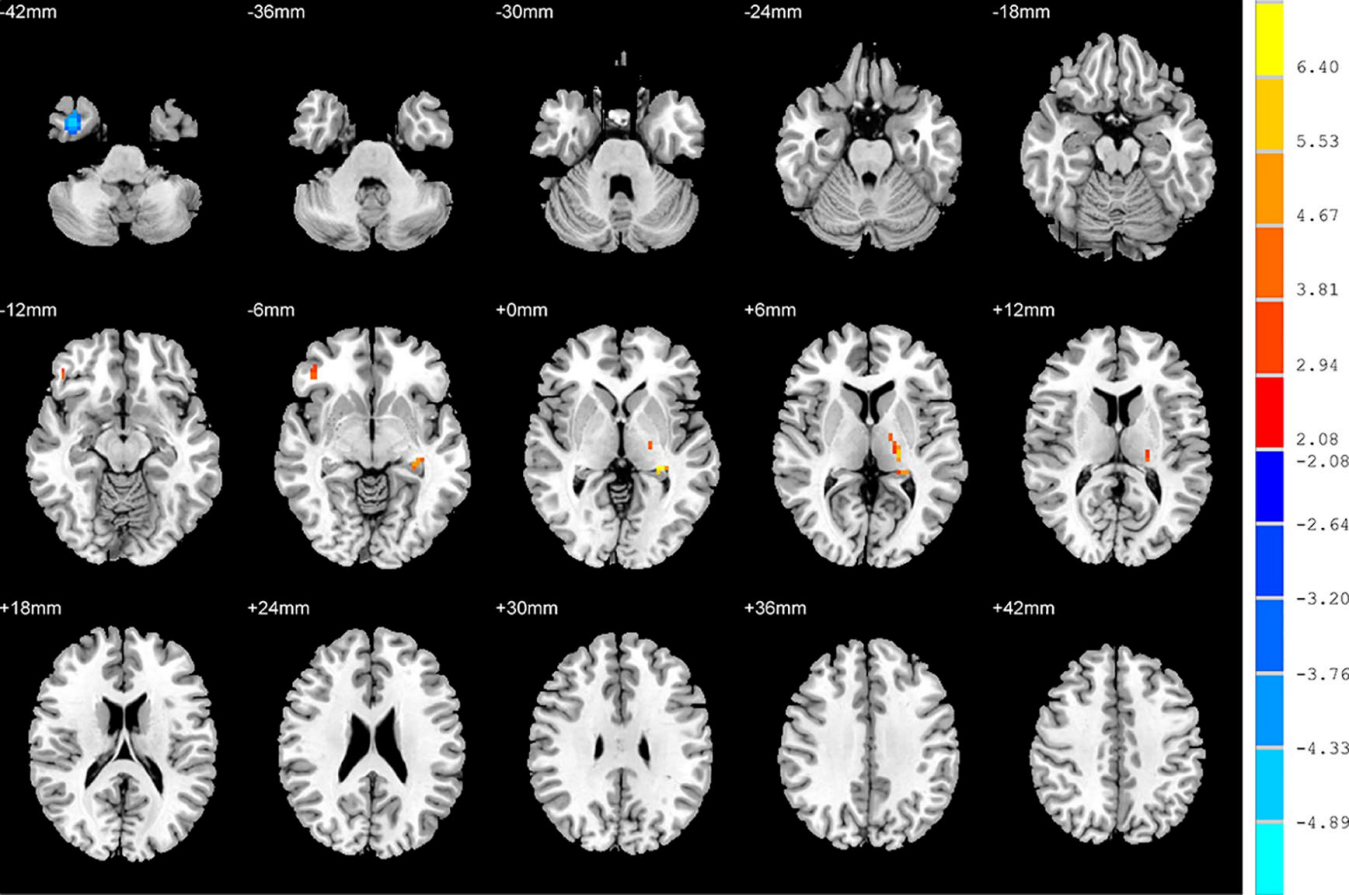
Brain regions showing differences in the amplitudes of low-frequency fluctuations between SD patients and the HC group. The colour bar signifies the t-value of the independent t-tests between the two groups with *p* < 0.05 and corrected for multiple comparisons using FDR.

Relative to the HC group, patients with NSD exhibited higher ALFF values in the left hippocampus, right inferior frontal orbital gyrus and left thalamus, and lower values in the right inferior temporal gyrus (*p* < 0.05, FDR corrected) (**Table 2** and **Figure 3**).

### ALFF: SD Patients versus HC

There were no significant differences in the ALFF between patients with SD and the HC group.

### FC: Group Differences

**Figure 4:**
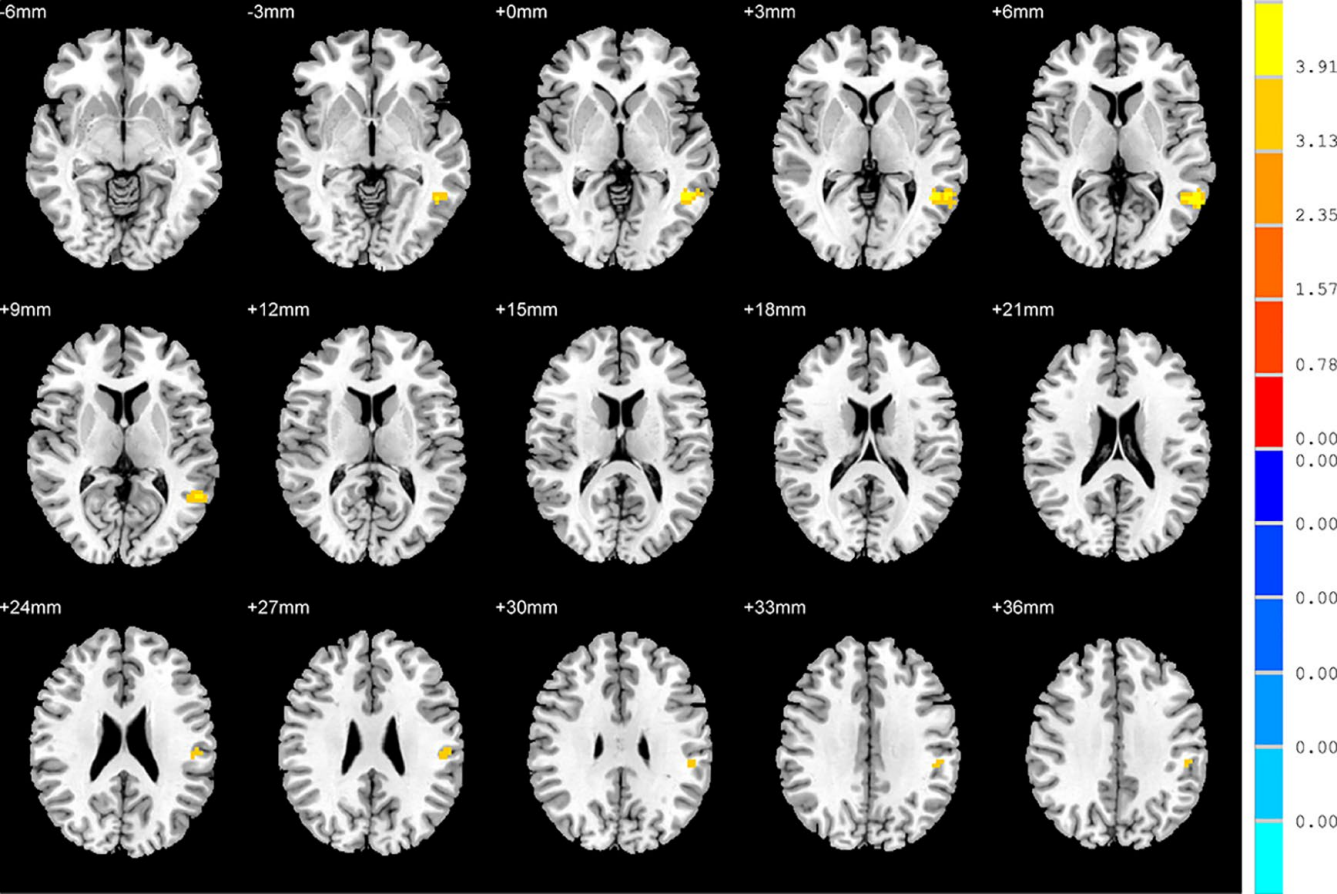
Brain regions showing differences in the functional connectivities among the three groups with age, sex and years of education as covariates. The colour bar signifies the F-value of the ANOVA analysis with *p* < 0.05 and corrected for multiple comparisons using GRF.

No significant findings were found among the three groups when using the three ROIs, including the right inferior temporal gyrus, left hippocampus and left thalamus, as the seeds. **Figure 4** shows the one-way ANOVA analysis of the FC values among the three groups. Significant group differences were detected in the right inferior frontal orbital gyrus with the left middle temporal gyrus, and the right inferior frontal orbital gyrus with the left inferior parietal cortex (*p* < 0.05, GRF corrected) (**Table 3** and **Figure 4**).

**Table 3:** Brain areas with significantly different functional connectivity for considered the right inferior frontal orbital gyrus as seed region towards voxels at whole brain among in the SD, NSD and HC groups.

Brain regions	MNI (x y z)			Cluster size	F/*t* -value
Three groups					
L Middle temporal gyrus	−48	−54	0	80	4.697^a^
L Inferior parietal cortex	−51	−36	57	85	4.641^a^
SD < NSD					
L Inferior parietal cortex	−51	−36	57	20	−4.852^b^
SD < HC					
L Middle temporal gyrus	−48	−54	0	60	−4.807^b^
L Inferior parietal cortex	−42	−42	57	68	−4.924^b^

SD, somatic depression; NSD, non-somatic depression; HC, healthy control; MNI, Montreal Neurological Institute; x, y, z, are the coordinates of the primary peak locations in the MNI space; F, statistical value of the peak voxel showing significantly differences in the functional connectivity with the right inferior frontal orbital gyrus seed among all the groups; t, statistical value of the peak voxel showing different functional connectivities with the right inferior frontal orbital gyrus seed in the SD compared to the NSD, or NSD and HC (p < 0.05, corrected for GRF correction); L, left; R, right.

a The F statistical value.

b The t statistical value.

### FC: SD versus NSD Patients

**Figure 5:**
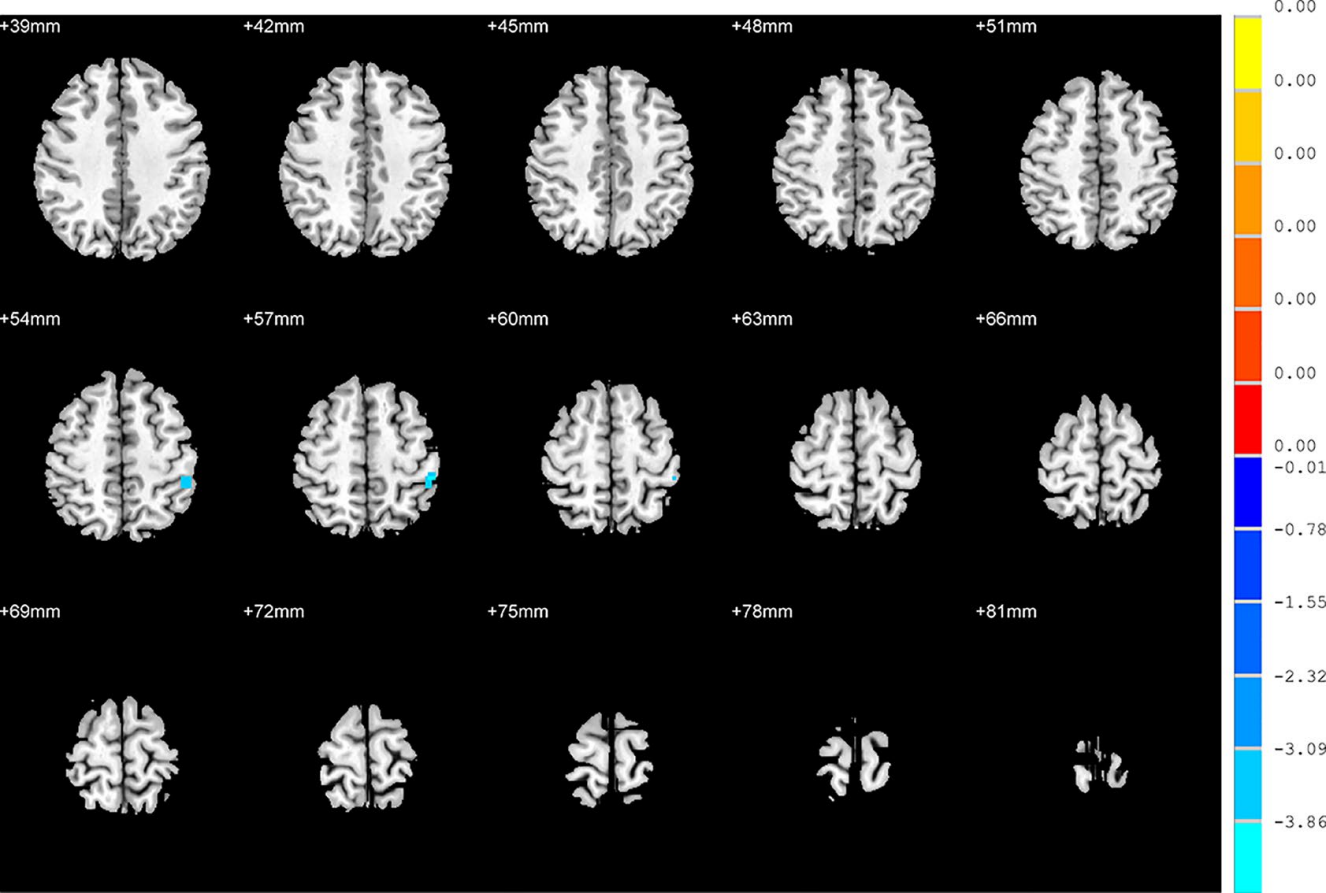
Brain regions showing differences in the functional connectivities between the SD patients and NSD patients. The colour bar signifies the t-value of the independent t-tests between the two groups with *p* < 0.05 and corrected for multiple comparisons using GRF.

Compared to the NSD patients, SD patients showed a significant decrease in the FC value in the right inferior frontal orbital gyrus with the left inferior parietal cortex (*p* < 0.05, GRF corrected) (**Table 3** and **Figure 5**).

### FC: SD Patients versus HC

**Figure 6:**
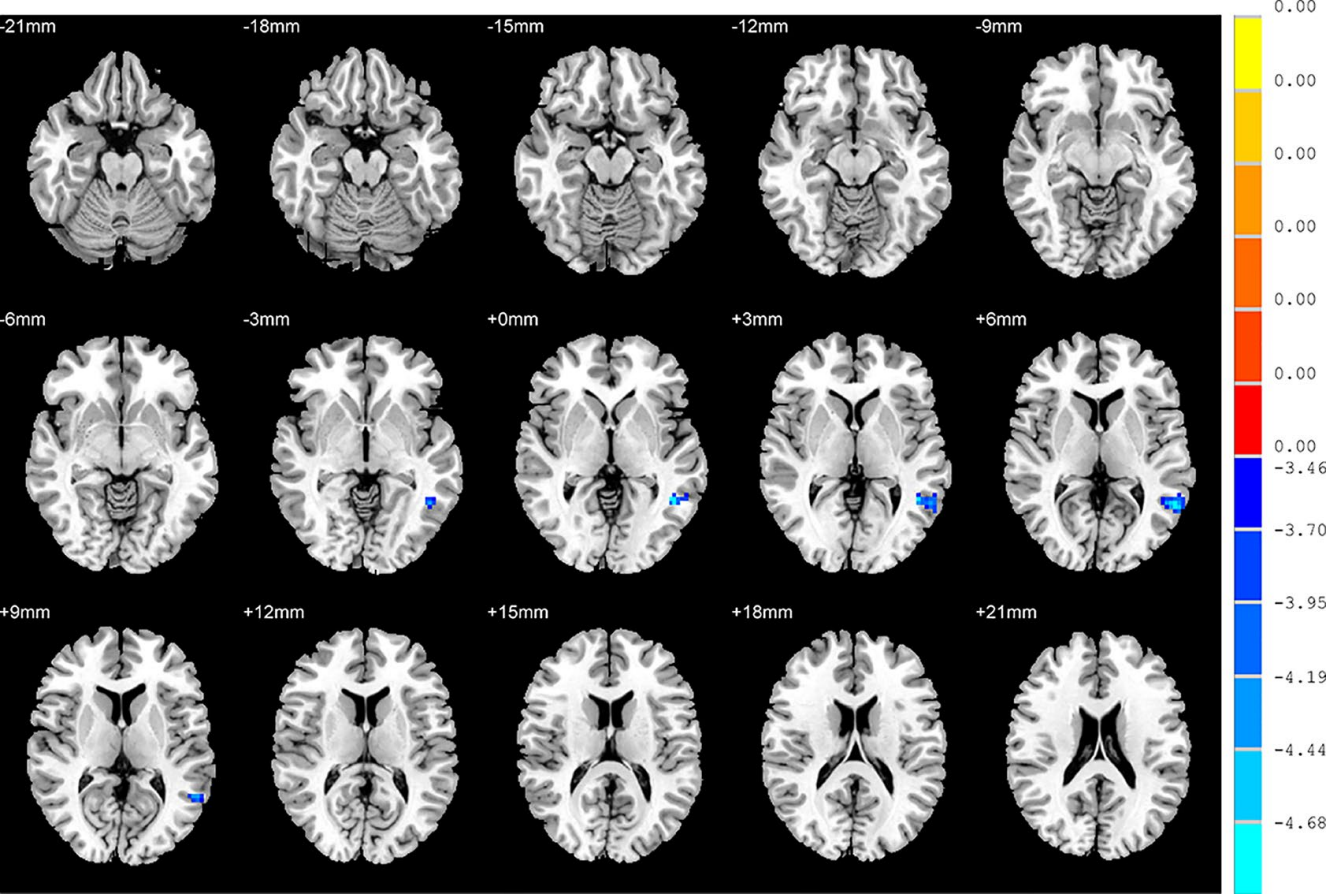
Brain regions showing differences in the functional connectivities between the SD patients and the HC group. The colour bar signifies the t-value of the independent t-tests between the two groups with *p* < 0.05 and corrected for multiple comparisons using GRF.

With regards to to the HC group, SD patients exhibited lower FC values in the right inferior frontal orbital gyrus with the left middle temporal gyrus, and the right inferior frontal orbital gyrus with the left inferior parietal cortex (*p* < 0.05, GRF corrected) (**Table 3** and **Figure 6**).

### FC: NSD Patients versus HC

There were no significant differences in the FC values between NSD patients and the HC group.

### Correlations between ALFF or FC and the, Somatic Symptoms Features

**Figure 7:**
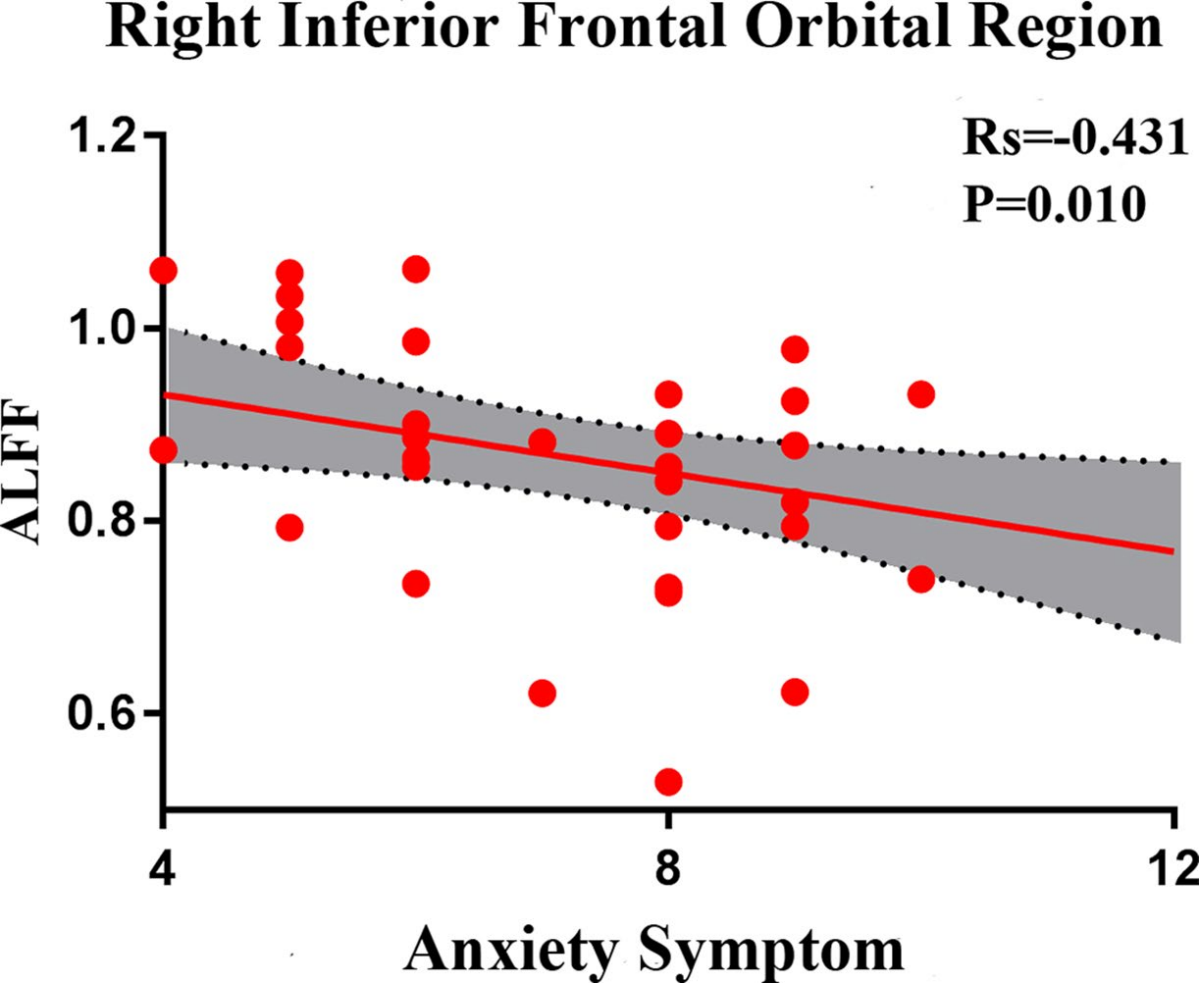
Negative correlation between the amplitudes of the low-frequency fluctuations of the right inferior frontal orbital gyrus and the severity of anxiety factor scores in the SD group. The X axis shows the score of the anxiety factors of the HAMD-17, and the Y axis is the amplitude of the low-frequency fluctuations of the right inferior frontal orbital gyrus.

In SD patients, the mean ALFF value for the right inferior frontal orbital gyrus was negatively correlated with the severity of anxiety factor scores (*r* = –0.431, p = 0.010, corrected, **Figure 7**). No other alterations in brain function demonstrated a significant correlation with the total HRSD scores and the three separate symptomatic factors scores.

## Discussion

The aim of this study was to examine the intrinsic activities and corresponding brain circuit alterations by comparing the whole-brain fMRI ALFF and the corresponding FC between MDD patients with and without somatic symptoms. Our analysis found that SD patients showed a significant increase in the ALFF for the right inferior temporal gyrus, and significant decreases in the left hippocampus, right inferior frontal orbital gyrus and left thalamus. There was also a significant decrease in the FC value between the right inferior frontal orbital gyrus and the left inferior parietal cortex. We found that areas with abnormal regional activity and the associated brain circuits were situated in the limbic-cortical systems, which were considered as important emotional or cognitive-related brain regions in depressed patients during the resting state. This finding confirmed our hypothesis that abnormal regional activity affects the associated brain circuits in SD patients. Moreover, the mean ALFF value for the right inferior frontal orbital gyrus was associated with the severity of anxiety symptoms. These results suggest that the SD phenomenon may be rooted in psychosocial forces (6). This may be an important aspect of the underlying mechanisms for the pathogenesis of SD in Chinese patients and around the world.

The right inferior frontal orbital gyrus is the upper part of the limbic lobe and a region of the orbitofrontal cortex. Recent evidence has indicated that the inferior frontal orbital gyrus is associated with a variety of brain functions, including memory-related emotions, self-awareness (43), cognitive regulation (44), memory, and reward (45). Yu et al. found that the orbitofrontal cortex is associated with interactions between insomnia and MDD and has an important role in the neuropathology of the comorbidity of insomnia and MDD (46). Our results were in line with these studies. Compared with NSD patients, we observed that the SD patients showed decreased ALFF values in the inferior frontal orbital gyrus, while the mean ALFF value of the right inferior frontal orbital gyrus was negatively correlated with the severity of anxiety symptoms.

Tozzi et al. found that the activation of the inferior frontal orbital gyrus was inversely correlated with the Beck’s depression inventory and HRSD total scores, which might be a relevant area for clinical symptoms of MDD (47). Yao et al. found that decreases in the regional homogeneity of the orbitofrontal cortex were related to cognitive deficits from depression (48). A previous study reported that the orbitofrontal cortex modulates the cognitive shift between internal and external environments and may affect anxiety through trait optimism (49).

Taken together with the above studies, the abnormal spontaneous neural activity in the inferior frontal orbital gyrus might lead to impaired mood regulation, insomnia, anxiety and lack of reward in SD patients, and exhibited a dissociation from the perceived effort required to obtain rewards (50). This could explain why SD patients have negatively biased attention in somatic symptoms, and do poorly in shifting attentional focus away from physical discomfort information (50). Thus, we speculate that the ALFF abnormality in the inferior frontal orbital gyrus may partially cause the pathogenesis of depression, anxiety and insomnia symptoms in SD patients.

In addition, the right inferior frontal orbital gyrus, right inferior temporal gyrus and left inferior parietal cortex are core components of the DMN (30). The DMN has been demonstrated to play a key role in the self-referential activities of MDD patients (51). Several studies have addressed the dissociation pattern of the DMN in MDDs (52, 53). In a study of a first-episode, drug-naive, somatization disorder, Su et al. found the right inferior temporal gyrus was associated with the severity of anxiety symptoms (54). In this study, we observed lower ALFF values in the right inferior frontal orbital gyrus and higher ALFF values in the right inferior temporal gyrus. We also observed increased FCs between the right inferior frontal orbital gyrus and the left inferior parietal cortex. The abnormal ALFF values in the right inferior frontal orbital gyrus were associated with the severity of anxiety symptoms in SD patients.

Our findings are in line with the above studies and indicate that abnormal activities in the inferior frontal orbital gyrus and right inferior temporal gyrus may reflect depressive moods, anxiety and insomnia symptoms in SD patients. Therefore, these abnormalities were used to determine the depressed state, which modulates the functional connectivity in DMN circuits (14, 51). These may be important pathomechanisms for dysfunctions in DMN circuits and could serve as potential indicators to quantitatively evaluate the severity of depression. Further studies are needed to verify this speculation.

Compared with NSD patients, SD patients showed lower ALFF values in the left hippocampus and left thalamus. The thalamus and hippocampus are important parts of limbic-cortical systems (55). The hippocampus is known to play an important role in memory consolidation and for the modulation of emotions and mood (56). The thalamus has been shown to play a crucial role in the awareness, sensory, motor and cognitive functions through the connectivity between its sub-nuclei and cortical and subcortical regions (57). It was reported by Jia et al. (58) that there are reduced fibre projections to the orbitofrontal and thalamus in depressed patients, which may disrupt the affective and cognitive functions to confer a heightened vulnerability and suicidal behaviour. Our study is consistent with the results of previous studies and indicates that the abnormal ALFF in the left hippocampus and left thalamus impaired the limbic-cortical systems, which combined with active intrinsic compensatory processes are seen to cause different somatic symptoms (59). These abnormal results may be associated with emotional and cognitive deficits in SD.

Finally, several limitations in this study must be acknowledged. First, the relatively small sample sizes may limit the generalization of our results. Second, we cannot exclude the influence of brain activity from medicine, although there was a minimum 2-week medication washout prior to the MRI scans (60). If all participant were required to be in an unmedicated state, there would be substantial practical and ethical hurdles. Third, although we instructed subjects to try and not think and to keep their eyes closed, the subjects still had thoughts or fell asleep during imaging. Finally, the sample sizes of the three groups were not equal. Although our data obey the normal distribution and homogeneity of variance, this fact may affect the effectiveness of the t-test statistics. In the future, the sample sizes should be increased.

In conclusion, we found that applying ALFF and FC analyses provide the impairment activity and connectivity in limbic-cortical systems and DMN circuits in SD patients. Our results suggest that abnormal regional alterations in the spontaneous neuronal activity may reflect a depressed state and should therefore be used as an index to modulate the functional connectivity during rest in SD patients. These may be important aspects of the underlying mechanisms for pathogenesis of SD at the neural level.

## Ethics Statement

This study was carried out in accordance with the recommen­dations of the Research Ethics Review Board of the Affiliated Nanjing Brain Hospital of Nanjing Medical University with written informed consent from all subjects. All subjects gave written informed consent in accordance with the Declaration of Helsinki. The protocol was approved by the Research Ethics Review Board of the Affiliated Nanjing Brain Hospital of Nanjing Medical University.

## Author Contributions

ZY and QL designed the study and wrote the protocol, and revised the paper. RY managed the literature research, analyses and wroted the first draft of the manuscript. ST, HL, YC, JS, YY and RZ collected experimental data and analyzed the results. All authors contributed to and have approved the final manuscript.

## Funding

This study was supported by grants of the National Natural Science Foundation of China (81571639, 61372032, 81871066), Jiangsu Provincial Medical Innovation Team of the Project of Invigorating Health Care through Science, Technology and Education (CXTDC2016004), Jiangsu Provincial key research and development program (BE2018609), Youth Medical Talent Project of Jiangsu Province (QNRC2016050), Youth Medical Talent Project of Affiliated Brain Hospital of Nanjing Medical University, Science and Technology Program of Nanjing (201605039, 201605041) and Nanjing Science and Technology Development Project (YKK15110, YKK16146). The authors thank the experimental conditions to be provided from National Key Clinical Department (2011–873), and Jiangsu province Key Clinical Discipline (2011–12).

## Conflict of Interest Statement

The authors declare that the research was conducted in the absence of any commercial or financial relationships that could be construed as a potential conflict of interest.
