# Supplementary material for: Abnormal Alterations of Regional Spontaneous Neuronal Activity in Inferior Frontal Orbital Gyrus and Corresponding Brain Circuit Alterations: A Resting-State fMRI Study in Somatic Depression
Source: Front Psychiatry. 2019 Apr 30;10:267. doi: 10.3389/fpsyt.2019.00267 (PMC6503088; doi:10.3389/fpsyt.2019.00267)
Supplement: Supplementary file 1 [file Table_1.doc]

**Supplemental Table 1**

List of antidepressant used in the SD group and NSD group.

| Variables | SD (n=35) | NSD(N=25) |
| --- | --- | --- |
| SSRI | 11 | 7 |
| SNRI | 8 | 5 |
| NaSSA | 3 | 2 |
| Medication naïve | 13 | 11 |

SD, somatic depression; NSD, non-somatic depression; SSRI, selective serotonin
reuptake inhibitor; SNRI, selective noradrenalin inhibitor; NaSSA, noradrenalin and specific serotonin antidepressant; Medication naïve, never be treated with any medicines before MRI scans.
