# Supplementary material for: Abnormal Alterations of Regional Spontaneous Neuronal Activity in Inferior Frontal Orbital Gyrus and Corresponding Brain Circuit Alterations: A Resting-State fMRI Study in Somatic Depression
Source: Front Psychiatry. 2019 Apr 30;10:267. doi: 10.3389/fpsyt.2019.00267 (PMC6503088; doi:10.3389/fpsyt.2019.00267)
Supplement: Supplementary file 2 [file Table_2.doc]

**Supplemental Table 2**

Brain areas with significantly different functional connectivity for considered the right inferior temporal gyrus, left hippocampus and left thalamus as seed region towards voxels at whole brain among in the SD, NSD and HC groups.

| Brain regions | MNI (x y z) | Cluster size | F*/t* -value |
| --- | --- | --- | --- |
| Functional connectivity with the right inferior temporal gyrus | | | |
| Three group | | | |
| L Postcentral gyrus | -39 -15 42 | 40 | 3.755^a^ |
| NSD<HC | | | |
| L Postcentral gyrus | -39 -12 48 | 40 | -4.703^b^ |
| Functional connectivity with the left hippocampus | | | |
| Three group | | | |
| None |  |  |  |
| Functional connectivity with the left thalamus | | | |
| Three group | | | |
| R Superior temporal gyrus | 69 -12 9 | 54 | 4.821^a^ |
| SD<HC | | | |
| R Superior temporal gyrus | 69 -12 9 | 54 | -4.930^b^ |

SD, somatic depression; NSD, non-somatic depression; HC, health control; MNI, Montreal Neurological Institute; x, y, z are the coordinates of primary peak locations in the MNI space; F statistical value of peak voxel showing significantly different functional connectivity in all the groups; t statistical value of peak voxel showing different functional connectivity in SD compared to NSD, SD compared to HC, or NSD and HC (p<0.05, corrected for GRF correction); L = left; R = right.

^a^ The F statistical value;

^b^ The t statistical value.
